# The Use of Eutectic Fe-Si-B Alloy as a Phase Change Material in Thermal Energy Storage Systems

**DOI:** 10.3390/ma12142312

**Published:** 2019-07-19

**Authors:** Jianmeng Jiao, Bettina Grorud, Caroline Sindland, Jafar Safarian, Kai Tang, Kathrine Sellevoll, Merete Tangstad

**Affiliations:** 1 Department of Materials Science and Engineering, Norwegian University of Science and Technology (NTNU), N-7491 Trondheim, Norway; 2 SINTEF Materials and Chemistry, N-7491 Trondheim, Norway

**Keywords:** Fe-Si-B, PCM, wettability, graphite, thermal cycle, energy storage material

## Abstract

Fe-26.38Si-9.35B eutectic alloy is proposed as a phase change material (PCM) as it exhibits high latent heat, high thermal conductivity, moderate melting point, and low cost. For successful implementation of it in the latent heat thermal energy storage (LHTES) systems, we investigate the use of graphite as a refractory material that withstands long-term melting/solidification in contact with the Fe-26.38Si-9.35B alloy. The PCM has been thermally cycled up to 1–4 times below and above its melting point at the temperature interval of 20 °C or 100 °C. It is observed that this eutectic alloy shows good thermal stability over a small temperature range of 1057–1257 °C. Some SiC and B_4_C solid precipitation will be formed at the top of the alloy. However, it does not seem to increase with time. The graphite crucible as a refractory material will produce a protective layer of SiC and B_4_C that will hinder the interaction between the PCM and the crucible. The small volume change during solidification will not break the graphite crucible during cycling. The chemical wear or dissolution of the crucible is negligible. It demonstrates the viability of Fe-26.38Si-9.35B alloy as a heat storage material in this type of container.

## 1. Introduction

The consumption of various forms of energy has increased over the past few decades, which leads to continuous growth in greenhouse gas emissions. With a rise in fuel prices, there has recently been a keen interest in producing and storing energies [1]. The pursuit for storage materials has led to a class of materials called phase change materials (PCMs), in which heat storage is carried out due to the fusion of latent heat or other phase changes. PCMs are chosen to undergo solid-liquid, solid-gas, liquid-gas, and solid-solid phase transformations, and typically most PCMs operate between solid-liquid phase transformation in thermal engineering applications, which is known as melting-solidification cycle [2]. The use of PCM storing energy can overcome the mismatch between the power supply and power demand [3].

Due to the advantages offered by latent heat thermal energy storage (LHTES), numerous low-temperature applications have been applied by the low-temperature PCMs [4]. However, most of the low-temperature PCMs have low thermal conductivity, chemical instability, and degradation after multiple thermal cycles. Hence, these disadvantages limit their application in thermal energy systems (TES) [5].

High-temperature PCMs based on eutectic metal alloys have, therefore, lately received considerable attention in the use of thermionic photovoltaic converter in the thermal energy storage systems due to their high thermal conductivity and good stabilities [6]. Khare et al. [7] found that metals such as aluminum, magnesium, silicon, and zinc, were useful for high-temperature heat storage in the temperature range of 400–700 °C. Particularly the alloys 88Al-12Si [8,9,10,11,12] and 60Al-34Mg-6Zn [13,14,15,16] were identified to have superior properties for PCMs. However, the maximum operating temperature is still below 1000 °C.

To develop a new generation material with ultra-high temperature energy storage beyond 1000 °C, Matthew (2015) [17] tried to use pure silicon as a PCM to develop a solar thermal propulsion system for micro-satellites. However, as the volume expansion of silicon was approximately 10%, it led to the PCM container breakage during solidification [18]. To decrease the pure silicon volume expansion, Datas et al. (2016) [19] proposed a novel PCM, silicon (Si)-boron (B) alloy based on the contraction of the silicon lattice on an alloy with boron [20]. Comparing with the regular PCMs, silicon and boron possess higher latent heat [21] and higher thermal conductivity. As a target high-temperature PCM, Si-B alloy has a desirable latent heat to affect the energy density and a moderate melting point at the eutectic point [19]. However, a strong penetration into the graphite crucible was observed by Homa et al. [22] as well as our unpublished experimental results [23]. The volume expansion of the Si-3.25B (mass %) alloy did not decrease much compared with the pure silicon. It is decided to develop a ternary X-Si-B alloy, aiming to minimize the interaction of the molten alloy with graphite container and to decrease the volume expansion of Si-B alloy upon solidification. Simultaneously, the latent heat of fusion should not drop dramatically. In this regard, iron was chosen as a third element to add to Si-B alloy for the first time to generate a eutectic Fe-Si-B alloy as a new PCM. Using FactSage commercial FTlite database, the chemical composition of the eutectic Fe-Si-B alloy was confirmed to consist of 64.27 mass % iron, 26.38 mass % silicon, and 9.35 mass % boron (Fe-26.38Si-9.35B) and it had a high latent heat at the melting temperature of 1157 °C. The thermal conductivity of the Fe-26.38Si-9.35B alloy was estimated by assuming the ideal mixture of pure elements of silicon [24], iron [25], and boron [26], as shown in Table 1. The estimated results showed that the increase in temperature resulted in a decrease in thermal diffusivity. The minimum thermal conductivity value being 30.6 W/(m∙K) at 1100 °C exhibits a high thermal conductivity. 

Isotropic graphite crucible is chosen as the potential PCM container for the Fe-26.38Si-9.35B alloy. This is a graphite material with isotropic structure and properties created through the cold isostatic press of micro particles. Isotropic graphite in the same direction has constant properties making it is ease of machining. The carbon atoms in the planes are covalently bonded in the graphite and it makes the material electrically conductive. Moreover, long time use is possible even at high temperatures exceeding 2000 °C in an inert atmosphere. With the exception of some strong oxidizers, it is chemically stable. Furthermore, its low thermal expansion and high thermal conductivity coefficients give it good thermal shock resistance. This study aimed to investigate the possibility to use the new Fe-26.38Si-9.35B PCM in graphite containers. Therefore, the phase formation of Fe-26.38Si-9.35B alloy, the interlayer between Fe-26.38Si-9.35B alloy and graphite crucible, the wettability behavior on graphite substrate and the penetration depth of Fe-26.38Si-9.35B alloy to graphite crucible after thermal cycling were researched systematically by experiments and phase equilibrium calculation. The obtained results established reliable data on thermophysical properties of Fe-26.38Si-9.35B alloy in both solid and liquid states around the melting point. It provides a technological basis for future implementing the PCM into reality in the LHTES application. 

## 2. Thermodynamic Properties of Fe-Si-B System

Thermodynamic calculations were carried out in order to determine the Fe-Si-B eutectic composition and its melting temperature. Furthermore, its phase formation in the cooling path and fusion latent heat were also predicted using FactSage software version 7.2 (Montreal, Canada and Aachen, Germany). Thermodynamic data were taken from the FTlite database.

The Fe-Si-B ternary system has three sub-binary systems, Fe-Si, Fe-B, and Si-B. The Fe-Si sub-binary system has five silicides. Fe_2_Si, FeSi, and Fe_3_Si_7_ are silicides with a small homogeneity range melting congruently. On the other hand, Fe_3_Si_2_ and FeSi_2_ silicides are involved only in solid state reactions. Fe_3_Si_7_ is a low-temperature form of the FeSi_2_ phase [27]. The Fe-B sub-binary system has two intermediate phases, Fe_2_B and FeB, and the latter melts congruently and has a narrow homogeneity range [28]. In addition, the Si-B sub-binary system has three intermediate phases: SiB_3_ a solid solution with a homogeneity range of 50.6–56.5 mass % boron, SiB_6_ stoichiometric compound, and SiB_n_ an extended solid solution between 82.6 and 91.9 mass % boron, respectively [20].

**Figure 1:**
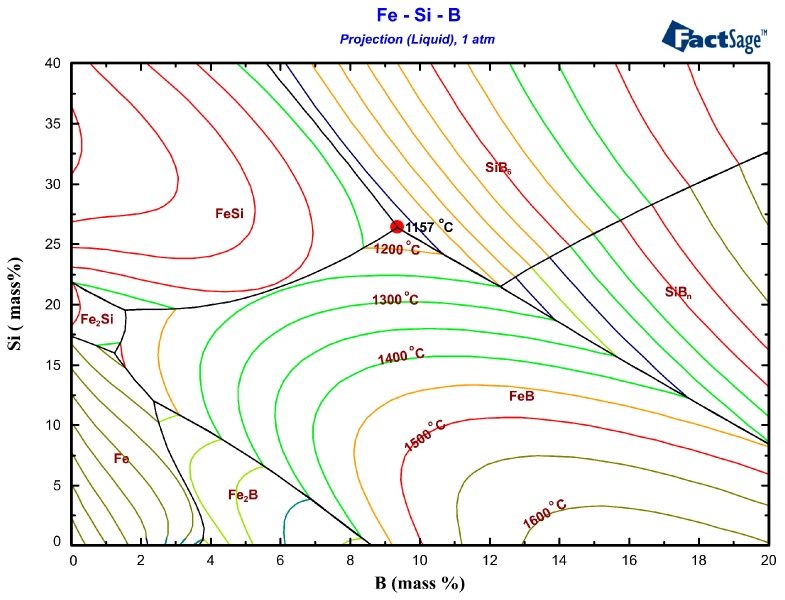
Projection of Fe-Si-B system, where red point represents the Fe-26.38Si-9.35B eutectic alloy (FTlite database).

Figure 1 illustrates the liquidus projection of the ternary Fe-Si-B system calculated by FactSage based on FTlite commercial database. The colored lines correspond to the isothermal curves. The nominal chemical composition of the eutectic Fe-26.38Si-9.35B alloy is presented at the red point, a eutectic reaction occurs when the temperature of the molten alloy decreases to 1157 °C, liquid → FeSi + FeB + SiB_6_. 

**Figure 2:**
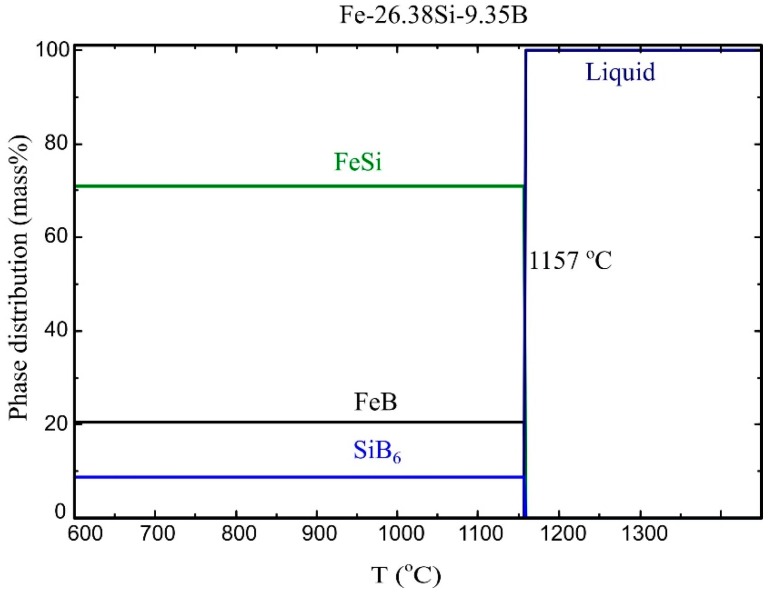
Cooling path of the Fe-26.38Si-9.35B alloy calculated by FactSage.

For illustrating the phase formation of the solidification process, the cooling path of the Fe-26.38Si-9.35B alloy from 1450 °C to 600 °C is shown in Figure 2. It is seen from the figure that three compounds, 70.9 mass% in FeSi, 20.4 mass% in FeB, and 8.7 mass% in SiB_6_ are formed when the temperature is below 1157 °C. 

**Figure 3:**
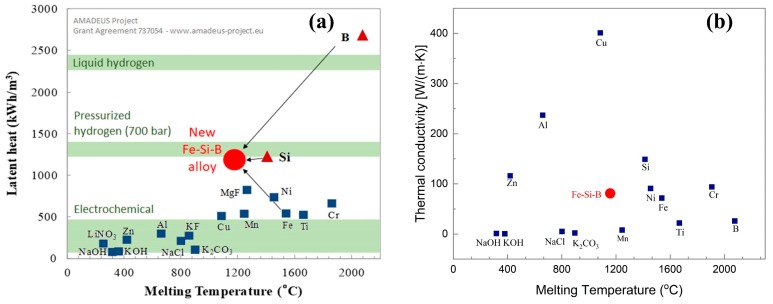
(**a**) Latent heat of fusion of different materials as a function of the melting temperature [19], (**b**) Thermal conductivity of different types of PCMs [29,30].

The latent heat of fusion of Fe-26.38Si-9.35B eutectic alloy was calculated by the FTlite database of FactSage 7.2. Figure 3a shows the relationship between the melting temperature and their latent heat for all the possible PCMs candidates. The red circle represents the latent heat of Fe-26.38Si-9.35B alloy. Comparing with all other possible candidates, the new Fe-Si-B alloy has a high latent heat (1250 kWh/m^3^) and moderate melting temperature (1157 °C). Additionally, silicon (second most abundant element on Earth) and iron (fourth most abundant element on Earth) are abundant and have low costs (silicon—1.7 $/kg) [19]. Furthermore, it shows a high thermal conductivity (Figure 3b). 

## 3. Experimental Procedure and Materials

The objective of this work was to investigate the phase formation in the Fe-26.38Si-9.35B alloy, the penetration depth of the molten Fe-26.38Si-9.35B alloy into graphite crucible after thermal cycling experiments, and the wettability behavior of Fe-26.38Si-9.35B particle on graphite substrate. This part describes the preparation method of master alloys, the graphite tube resistance furnace, the sessile drop furnace, and the characterization methods according to the research topic.

### 3.1. Materials

In this study, commercial pure solar grade silicon in granule form from fluidized bed reactor (FBR) process, boron powder (99.9 mass %), and iron powder (99 mass %) are chosen as the raw materials. The impurities in boron powder analyzed by inductively coupled plasma-mass spectrometer (ICP-MS) are summarized in Table 2. It is seen from Table 2 that iron is the main impurity in boron powder, other contaminants are less than 51 ppm mass and their influence on the Fe-Si-B alloy is negligible.

**Figure 4:**
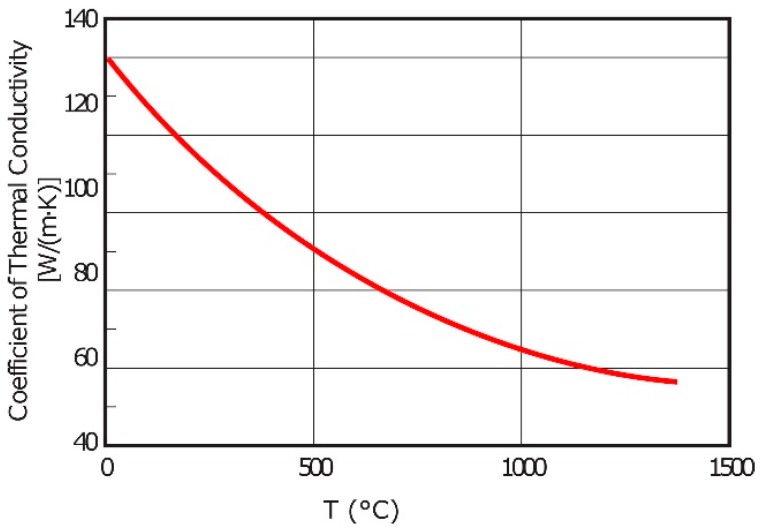
Thermal conductivity coefficient of the used graphite crucible vs. temperature.

Two different crucibles of graphite and alumina (Al_2_O_3_) were used for the experiments. A dense graphite crucible with an outer diameter of 22 mm and 15 mm inner diameter was machined from a block of isostatically pressed graphite and further used for thermal cycle experiments in a resistance furnace. Al_2_O_3_ crucible was used to make a Fe-26.38Si-9.35B master alloy. The properties of the graphite and Al_2_O_3_ crucibles are summarized in Table 3. Especially, the function of the thermal conductivity coefficient of the used graphite crucible with temperature is shown in Figure 4.

The eutectic Fe-26.38Si-9.35B master alloy was prepared as follows. The iron, silicon, and boron particles were weighed in an amount of 150 g and layered by descending melting temperature, then melted in the Al_2_O_3_ crucible in an induction furnace under argon. The Al_2_O_3_ crucible was placed inside a larger graphite crucible. The raw materials were melted through heating to 1700 °C, holding it for 1 h at this temperature for complete melting and homogenization. The solidified master alloy was then taken from the Al_2_O_3_ crucible and crushed in a mortar into pieces. The chemical composition of the eutectic Fe-26.38Si-9.35B master alloy was analyzed by ICP-MS. Both the detected and normalized content of silicon, iron, boron, aluminum, and manganese, in addition to the nominal composition of the alloy calculated by FactSage, are given in Table 4. Two master alloys were made in Al_2_O_3_ crucible, so we labeled them as Fe-Si-B-1, and Fe-Si-B-2. It is seen from Table 4 that the impurity contents of the aluminum and manganese are low. The Fe-Si-B-2 master alloy is closer to the nominal composition compared with the Fe-Si-B-1.

### 3.2. Thermal Cycle Experiments of Fe-26.38Si-9.35B PCM

**Figure 5:**
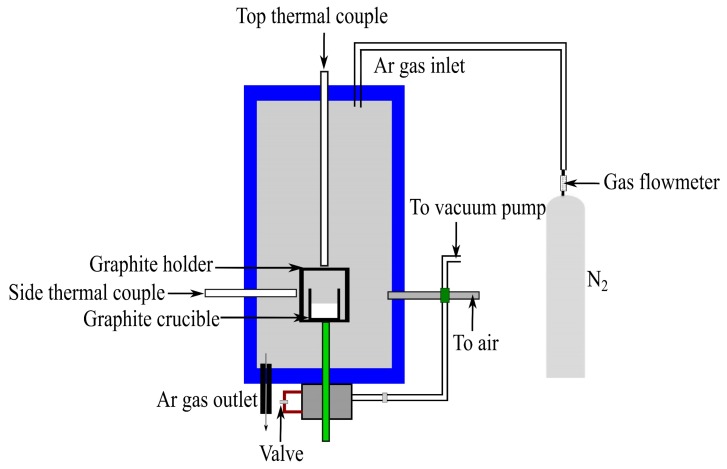
Schematic of the resistance vertical graphite tube furnace [31].

The thermal cycle experiment was carried out using 6–12 g of the Fe-26.38Si-9.35B master alloy in a graphite crucible in a vertical graphite tube furnace. The heat is supplied by resistive heating, in which the passage of an electric current through a graphite element produces heat. Two different types of thermocouples, B-type in the side and C-type in the top, were used to measure the furnace temperature. The thermocouples were calibrated by melting pure copper. Meanwhile, the thermogradient in the graphite tube furnace is regularly checked before the experiment and the temperature variation within the sample is less than 2 °C. This deviation is within the accuracy level for the thermocouple. The schematic of the furnace is shown in Figure 5. 

**Figure 6:**
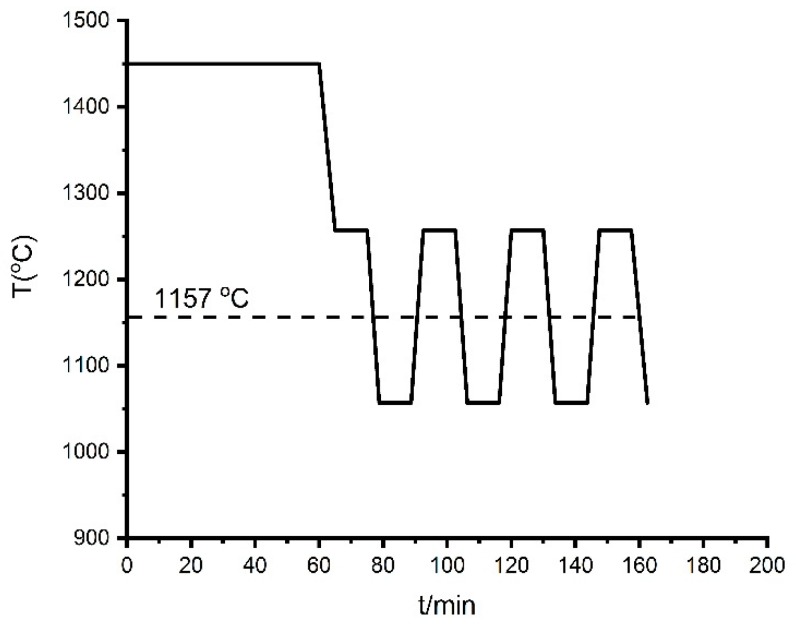
A graphic representation of the typical thermal cycle experiment.

To investigate the thermal cycle behavior of the Fe-26.38Si-9.35B PCM, we exposed the alloy to its melting point, re-melted and solidified in 1-4 thermal cycles at the temperature interval of 1157 ± 20 °C and 1157 ± 100 °C in argon atmosphere. Figure 6 presents one of the applied temperature profiles; the furnace was heated to 1450 °C, then it was held for 1 h at 1450 °C for homogenization. After that, the thermal cycle at 1157 ± 100 °C was initiated with a holding time of 10 min at the top and bottom of each interval.

### 3.3. Wetting Experiment

**Figure 7:**
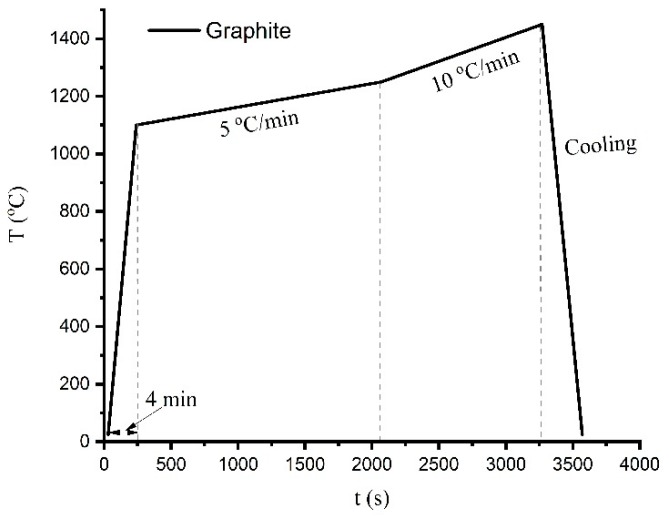
The temperature profile of the Fe-26.38Si-9.35B sample on the graphite substrate in the wetting test.

The wetting experiment was conducted by a sessile drop method on graphite substrate in a dedicated resistance furnace. A small piece of Fe-26.38Si-9.35B master alloy was chosen and placed on the graphite substrate. Heating was performed in a vacuum of 10^−1^ mbar up to 1100 °C in 4 min. Next, the alloy was heated to 1450 °C with a rate of 20 °C/min and, finally, it was cooled down to room temperature with a natural cooling rate. The temperature profile is presented in Figure 7.

### 3.4. Structural Characterization

**Figure 8:**
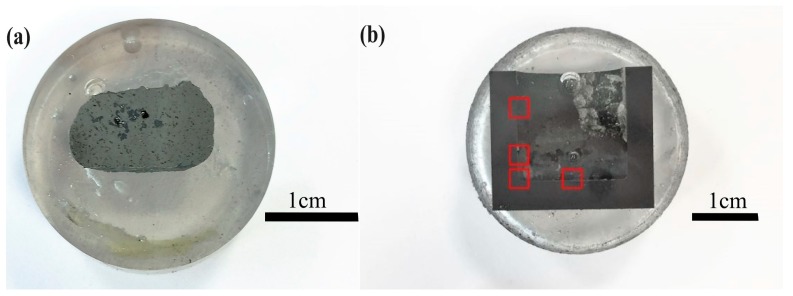
Graphite crucible and Fe-Si-B sample after being mounted, ground, and polished. (**a**) the Fe-26.38Si-9.35B alloy after thermal cycle experiment, (**b**) the graphite crucible after thermal cycle experiment, the red rectangles represent the positions that are analyzed by optical microscopy.

The solidified alloy in the graphite crucible was cut with a diamond cut-off wheel. Then, the metallographic preparation was performed by a stepwise electronically controlled grinding and polishing machine. The Fe-26.38Si-9.35B sample and crucible after being mounted, ground, and polished are shown in Figure 8. It is observed that the sample and graphite crucible is easily separated.

The microstructures were studied by electron probe micro-analyzer (EPMA) (JEOL JXA 8500F, Germany) and scanning electron microscope (SEM) (Zeiss Supra, 55 VP, Germany). The SEM was supplied with secondary electron (SE) contrast and backscattered electron (BSE) detector at an acceleration voltage of 15 or 20 kV and a working distance of 10 mm. The inner surface of the crucible was analyzed through optical microscopy (OM) (Leica MEF4M, Germany), and images were taken from three positions at the left wall, the left corner, and the bottom of the sample, as seen in Figure 8b. Moreover, the industrial computed tomography (CT) (XT H 225 ST, Nikon, Japan) scanning technique was also used to produce 2D cross-sectional images of the alloy to qualify the densification of the solidified alloy. The chemical composition of the formed phase was determined by wavelength dispersive X-ray spectroscopy (WDS) technique. Phase analysis was also performed using an X-ray diffractometer (XRD) (Bruker D8 A25 DaVinci, Germany) with Cu Kα radiation, 35–105 deg diffraction angle, 0.013 deg step size. The diffraction peak patterns were analyzed using DIF-FRAC.EVA software (Bruker AXS GmbH, Karlsruhe, Germany) and the JCPSD-database PDF-4 + 2018 RDB. 

## 4. Results and Discussion

### 4.1. Phase Formation in the Fe-26.38Si-9.35B Alloy

**Figure 9:**
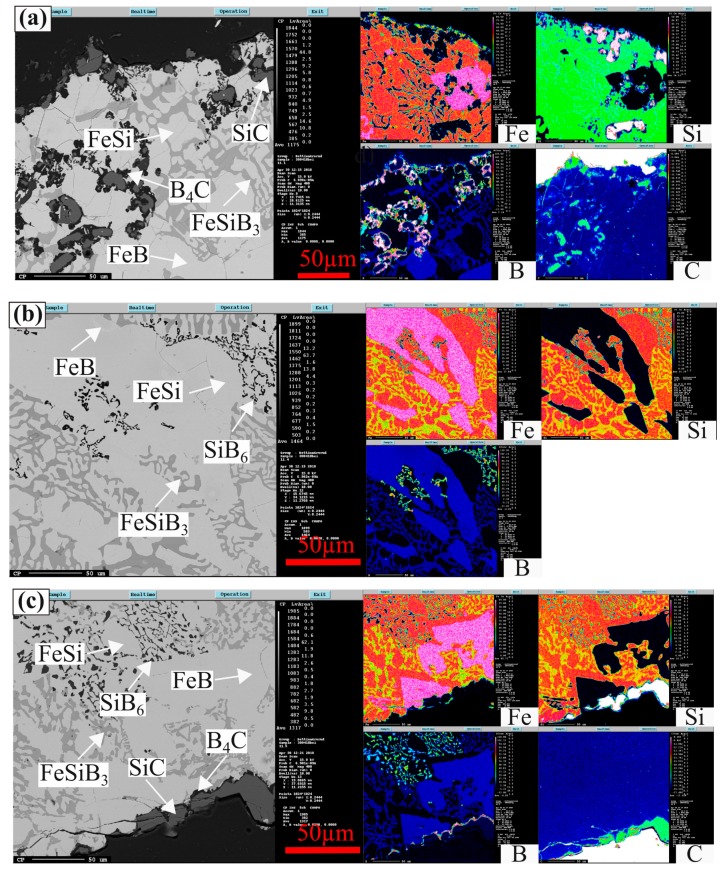
EPMA images of Fe-26.38Si-9.35B alloy after two times melting/solidification cycles at 1157 ± 20 °C: (**a**) at the top, (**b**) at the center, and (**c**) at the bottom.

The formed phases are detected in the Fe-26.38Si-9.35B alloys after 1–4 melting/solidification process, which contains FeSi, FeB, SiB_6_, SiC, B_4_C, and FeSiB_3_. Figure 9a–c present the distribution of elements measured by EPMA for the Fe-26.38Si-9.35B alloy at top, center, and bottom three different positions, in which the alloys have be subjected to two thermal cycles at 1157 ± 20 °C It is apparent from Figure 9a–c that FeSi phase is determined as the metal matrix, and FeSiB_3_ is identified as a new phase by WDS analysis (Table 5). The most interesting aspect of this figure is that FeSi and FeB phases can only be distinguished in the element distribution pictures, instead of BSE pictures. Furthermore, the top position of the sample (Figure 9a) shows that some SiC particles exist in the alloy and the SiC particles are decorated with B_4_C phases. In the bulk of the sample (Figure 9b) the SiC and B_4_C phases are not detected. Hence, five phases are present at the top position of the metal, four phases in the bulk of the alloy, and six phases in the bottom area towards the graphite crucible.

**Figure 10:**
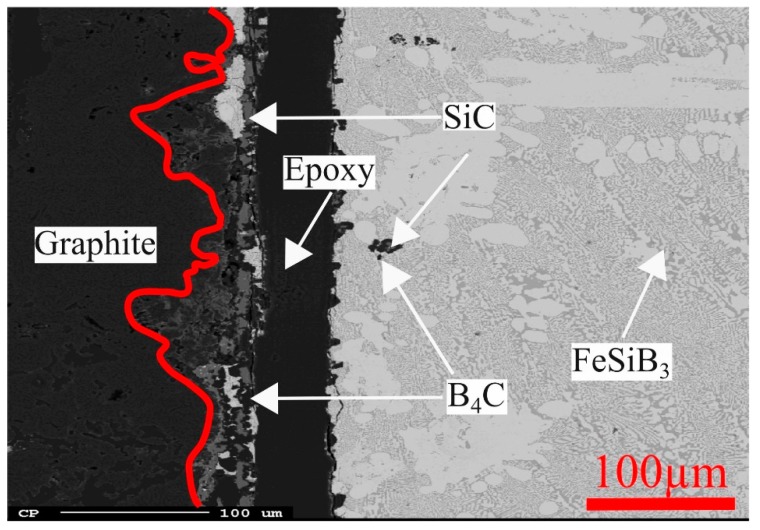
The distribution of phases at the interface between Fe-26.38Si-9.35B alloy and the graphite crucible after four thermal cycles at 1157 ± 100 °C.

A continuous SiC and B_4_C layer was generated between the Fe-26.38Si-9.35B alloy and graphite crucible. A typical EPMA image on the interface between graphite crucible and Fe-Si-B alloy after four thermal cycles at 1157 ± 100 °C is shown in Figure 10. Combined with Figure 9c, we know that two continuous barrier layers, a thicker outer layer of SiC and a thinner B_4_C inner layer, are formed at the interface between the PCM alloy and graphite crucible.

All phases in the samples after thermal cycles were analyzed by WDS in EPMA, and each phase was measured at least twenty points in the samples, the chemical component of FeSi, FeSiB_3_, FeB, and SiB_6_ are summarized in Table 5.

**Figure 11:**
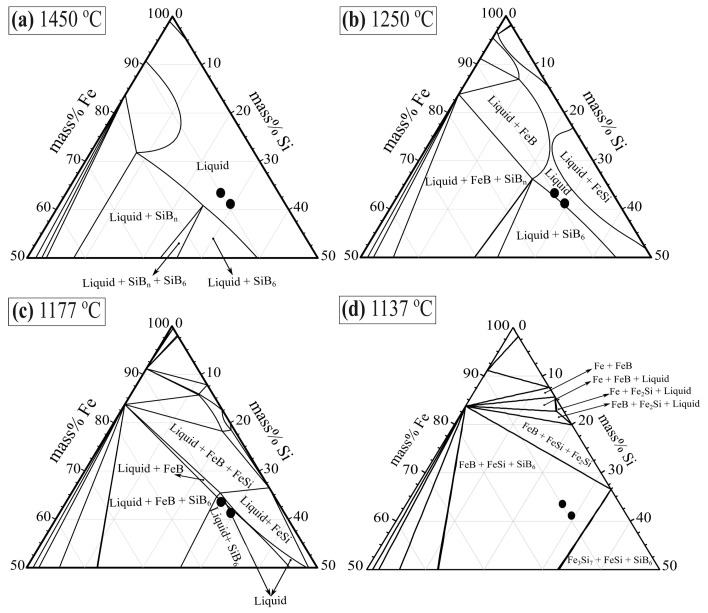
Isothermal cross-section of the ternary Fe-Si-B system at the iron range 50–100 mass % calculated by FactSage at temperatures of (**a**) 1450 °C, (**b**) 1250 °C, (**c**) 1177 °C, and (**d**) 1137 °C.

Figure 11 presents the isothermal cross-section of Fe-Si-B ternary system within 50–100 mass % iron at the temperature range of 1137–1450 °C. The two black points represent the chemical composition of the two master alloys produced and analyzed by ICP-MS, used in the thermal cycle experiments and measurements of thermophysical properties. It is seen from Figure 11 that they are both in the liquid area at 1250–1450 °C, and then transferred to the liquid + SiB_6_ phase area when the temperature decreases to 1177 °C, and through further cooling, they should reach the FeB + FeSi + SiB_6_ phase region at 1137 °C. The thermal cycle experiment performed at the temperature range of 1137–1177 °C has a phase composition between liquid + SiB_6_ and FeB + FeSi + SiB_6_ phase area. The solidification and melting process is undertaken between the liquid phase and FeB + FeSi + SiB_6_ phases area when the temperature interval is changed between 1057 °C and 1257 °C.

**Figure 12:**
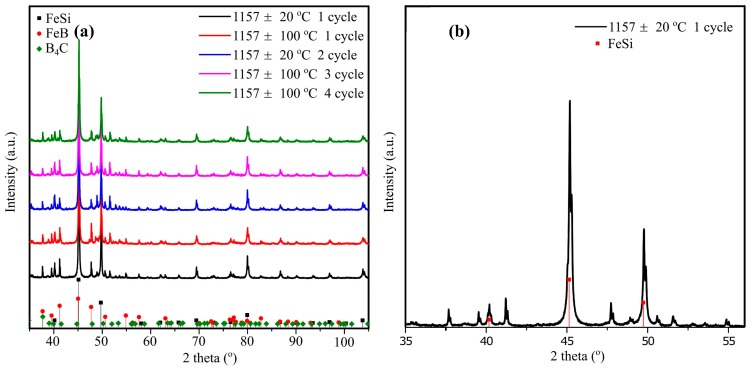
(**a**) XRD patterns of Fe-26.38Si-9.35B alloys with different temperature intervals and thermal cycles. (**b**) An expanded view of the FeSi XRD spectra at the scan range of 35–55°, showing a clear peak shift towards left.

Compared with the experimental results, FeSi, FeB, and SiB_6_ are expected from the calculated diagram. It is seen from Table 5 that ~11 at% boron was dissolved to the FeSi phase and formed an interstitial solution, which was verified by the XRD results, where the offset of the FeSi peak towards left (Figure 12b). Simultaneously, only small traces of silicon (0.23 at %) was detected in the FeB phases. 

FeSiB_3_ is not expected from the calculated phase diagram. The measured average chemical composition of the new phase FeSiB_3_ was 22.0 at % Fe, 20.6 at% Si, and 57.3 at %. Neamţu et al. [32] documented that only Fe_3_Si and Fe_2_B phases existed in the Fe-11.6Si-1.1B amorphous powder after heated up to 900 °C in the DSC furnace. Moreover, Tong et al. [33] prepared the nanocrystalline FeBSi alloy specimens in a vacuum furnace by heating the amorphous FeBSi ribbons, and α-Fe(Si) solid solution and Fe_2_B phases were confirmed by XRD analysis. They also did not detect any ternary Fe-Si-B compounds. At present, FeSiB_3_ is assumed to be a new observed phase, which is different with the other three ternary compounds Fe_5_Si_2_B, Fe_4.7_SiB_2_, and Fe_2_Si_0.4_B_0.6_ described by Aronsson [34]. Further investigation should be performed to confirm the FeSiB_3_ lattice structure.

**Figure 13:**
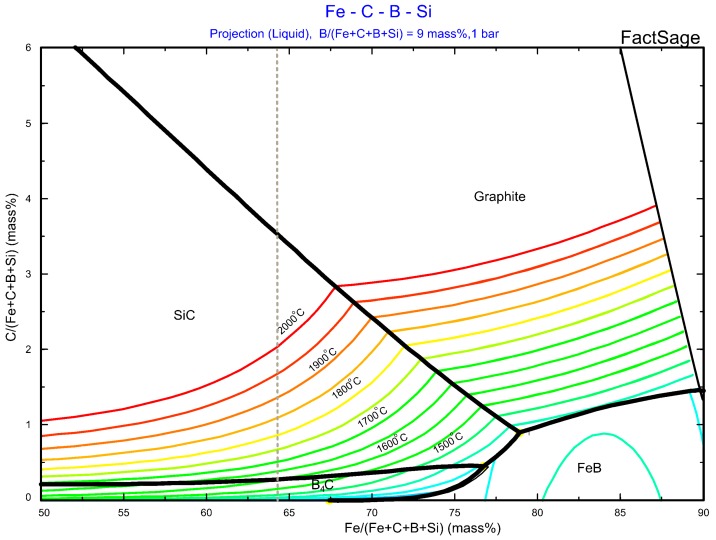
Liquidus projection of Fe-Si-9mass %B-C phase diagram.

The produced Fe-26.38Si-9.35B eutectic alloy was melted in graphite crucible and, hence, the carbon would be dissolved into the melts. The dissolved carbon will change the composition of the alloy. To investigate the influence of carbon on the phase formation in Fe-26.38Si-9.35B alloy, the liquidus projection in the Fe-Si-9B-C (mass %) alloy was calculated using the SINTEF database [35], as shown in Figure 13.

The primary crystallized phases are SiC, B_4_C, FeB, and graphite. SiC and graphite phases are stable at the high-temperature and higher carbon range, whereas, B_4_C and FeB phases are stable at the low-temperature and at lower carbon range. When the molten Fe-Si-B alloy equilibrates with graphite crucible, the carbon solubility increases with increasing temperature at a constant iron content or with the increase of iron content at a constant temperature. The grey dashed line represents the constant iron content of 64.2 mass %, which shows that SiC coexists with Fe-26.38Si-9.35B melts at temperatures above 1600 °C. However, it reacts with boron to form B_4_C when the temperature decreases to the B_4_C phase area. The maximum operating temperature of the present work is 1450 °C, which is lower than 1600 °C. Hence, the molten Fe-26.38Si-9.35B alloy should be equilibrated with B_4_C. This is confirmed by the experimental results, i.e., the formation of B_4_C layer at the interface (Figure 10).

According to Figure 13, the carbon solubility in the Fe-26.38Si-9.35B alloy is at the range of 0.1–0.3 mass % at the temperature range of 1500–1600 °C, which means that carbon dissolved into the melts is low. As the temperature decreases during cooling, the dissolved carbon is precipitated to SiC particle. With the decreasing temperature, B_4_C will precipitate at 1396 °C [36]. However, these phases are only found at the top position of the alloy. They are not detected in the bulk of the sample. According to the calculation s by FactSage, the density of the molten Fe-26.38Si-9.35B alloy, B_4_C, and SiC is 4.3836 g/mL, 2.52 g/mL, and 3.217g/mL, respectively. This means that B_4_C and SiC particles will float to the top of the molten Fe-Si-B alloy in the melting/solidification process due to their lower density. 

### 4.2. Effect of Thermal Cycling 

**Figure 14:**
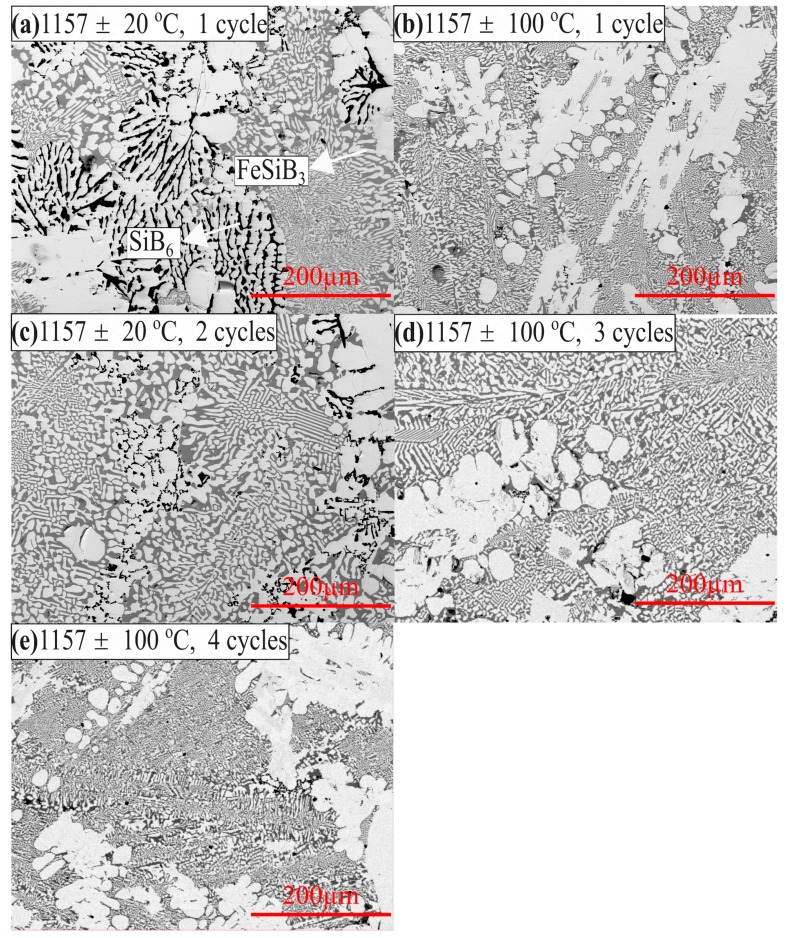
Backscattered electron images at different thermal cycles in the center position of the samples. (**a**) 1 cycle at 1157 ± 20 °C, (**b**) 1 cycle at 1157 ± 100 °C, (**c**) 2 cycles at 1157 ± 20 °C, (**d**) 3 cycles at 1157 ± 100 °C, (**e**) 4 cycles at 1157 ± 100 °C.

The stability of the phases under solid-liquid cycles near the melting point in a PCM is crucial to obtain a long lifecycle. Therefore, thermal cycle experiments were performed to identify the stability of the observed phases in the Fe-Si-B alloy inside the graphite crucible during solidification and melting process, where the thermal cycle and the temperature range was designed as 1–4 times at 1157 ± 20 °C and 1157 ± 100 °C. The morphologies of the samples in the bulk of the samples after the thermal cycle experiments are shown in BSE images in Figure 14a–e. By comparison, the BSE images show that the structures in the Fe-26.38Si-9.35B alloys after thermal cycle experiments are similar, which are consisted of FeSi, FeB, FeSiB_3_, and SiB_6_.

The samples after thermal cycle experiments were also analyzed by XRD and are shown in Figure 12. It is seen from the figure that the FeSi, FeB, and B_4_C phases were verified by XRD. However, the SiC and SiB_6_ phases were not detected. The FeSiB_3_ phase was not found in the XRD database (PDF-4 + 2018 RDB). The five XRD spectrums represent five different thermal cycle conditions, which are similar. It substantiates the phase stability in the Fe-26.38Si-9.35B alloy after 1–4 thermal cycles during melting/solidification process.

**Figure 15:**
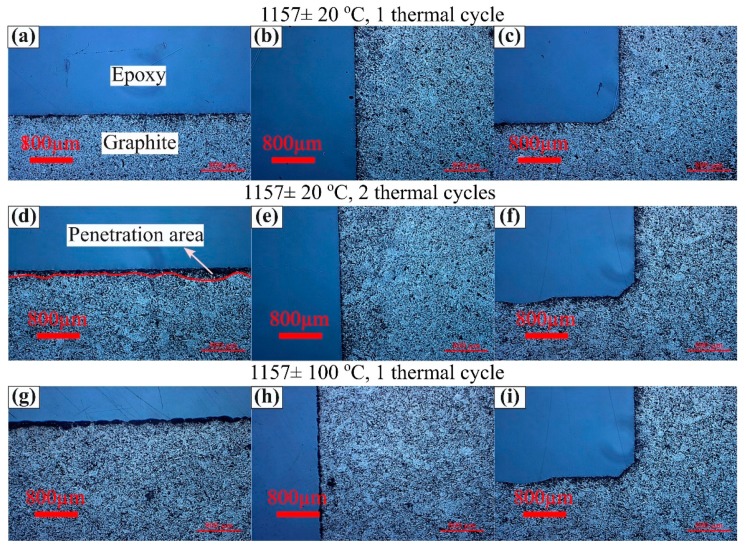
Images of the contact area of the alloy and the graphite crucible at (**a**,**d**,**g**) bottom of the crucible, (**b**,**e**,**h**) left wall of the crucible, and (**c**,**f**,**i**) left corner of the crucible.

The penetration depth of the alloy into the graphite crucible after thermal cycle experiments was measured at the bottom, center, and side positions of the crucible by optical microscopy and is presented in Figure 15. The crucibles experienced little penetration from the molten Fe-26.38Si-9.35B alloy. The penetration depth in these samples was measured at higher magnifications. It is found that they are to be 60–180 µm at the bottom, 40–120 µm at the corner, and 40–100 µm at the wall. The similar measurements were conducted from the Si-3.25B/graphite system. The median penetration depth was found to be 1150 µm at the bottom, 1150 µm at the corner, and 830 µm at the wall [23]. It indicates that the penetration depth of the molten Fe-26.38Si-9.35B into the graphite crucible is negligible compared with molten Si-3.25B alloy. Therefore, the graphite material can be used for long-term thermal cycles without breakage.

### 4.3. Wettability of Graphite Substrate by the Molten Fe-26.38Si-9.35B 

**Figure 16:**
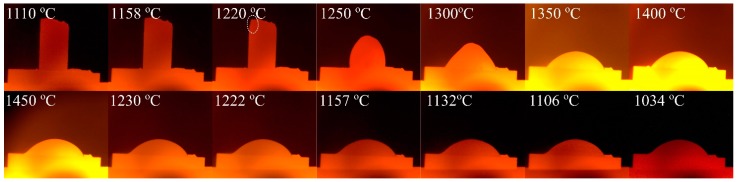
Images of Fe-26.38Si-9.35B droplets on the graphite substrate under vacuum in the sessile drop wettability test.

The Fe-26.38Si-9.35B alloys could easily be detached from the crucibles after thermal cycle experiments or during the cutting process. To explain this phenomenon, an investigation of the wettability property of the Fe-26.38Si-9.35B alloy on graphite substrate is done on graphite substrate. It is beneficial to obtain a proper view of the crucible material. The images of the sample taken during the wetting test are presented in Figure 16.

Figure 16 shows that the Fe-26.38Si-9.35B particle started to melt at about 1220 °C, which is shown as the white dotted circles in the Figure. This is somewhat higher than the calculated eutectic temperature from the FactSage calculation of 1157 °C. It may be caused by the oxide layer existed at the surface of the Fe-26.38Si-9.35B particle. The complete melting of the alloy on the graphite substrate was observed at 1234 °C. The final contact angle decreased to 31° at 1450 °C, showing high wettability with graphite materials. 

Cao er al. [37] measured the contact angles of Fe-5.3Si-3B alloy on SiC substrate under vacuum. The contact angle of 100° on SiC substrate at 1150–1360 °C had a considerable difference with our experimental result of 31° on graphite at 1450 °C. It is known that the wetting angle of pure iron on graphite exhibited good wetting at 1300–1500 °C, with 0–66° under different atmospheres [38,39,40,41]. However, it increased from less than 90° to around 140° when carbon content increased in the melt [39]. In the study performed by Rubio et al. [42], the Fe-14.1Si alloy was found to be much lower, reaching 38° at 1550 °C, which had a similar angle with our experimental result.

The wetting tests show that the eutectic Fe-26.38Si-9.35B alloy displays good wetting behavior on the graphite substrate, which will make the Fe-26.38Si-9.35B alloy adhering to the graphite instead of detaching. Therefore, the detaching phenomenon in the experiments may be caused by other unknown reasons and it will be further investigated. Though the wettability towards the transformed C-crucible is high, it was still penetrating the crucible less than Si-B alloys, which had been observed by our work in the lab [23]. The melting point was observed around 1220 °C, which was higher than the theoretical melting point of 1157 °C. 

### 4.4. Pore Formation in the Solidified Fe-26.38Si-9.35B Alloy

**Figure 17:**
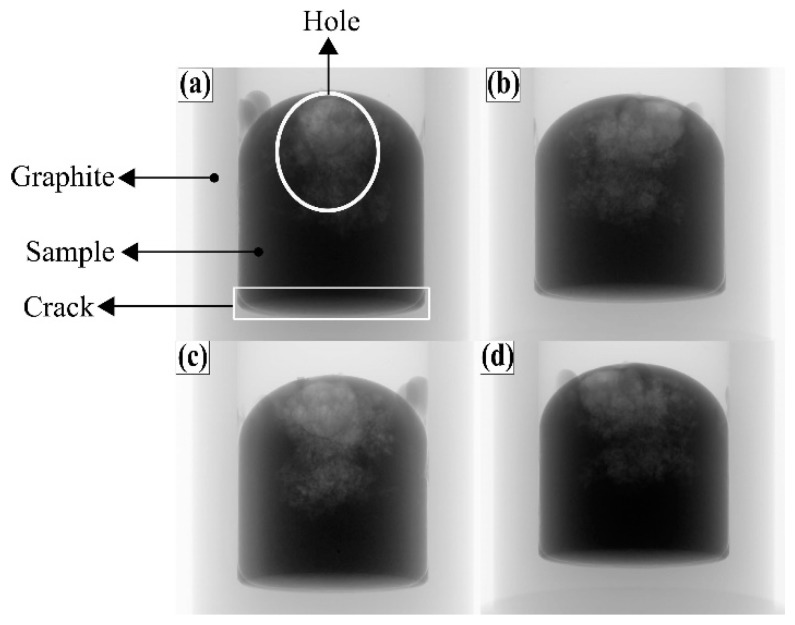
2D-CT images of graphite crucible with solidified Fe-26.38Si-9.35B alloy at different angle position. (**a**) front view, (**b**) right side view, (**c**) rear view, and (**d**) left side view.

**Figure 18:**
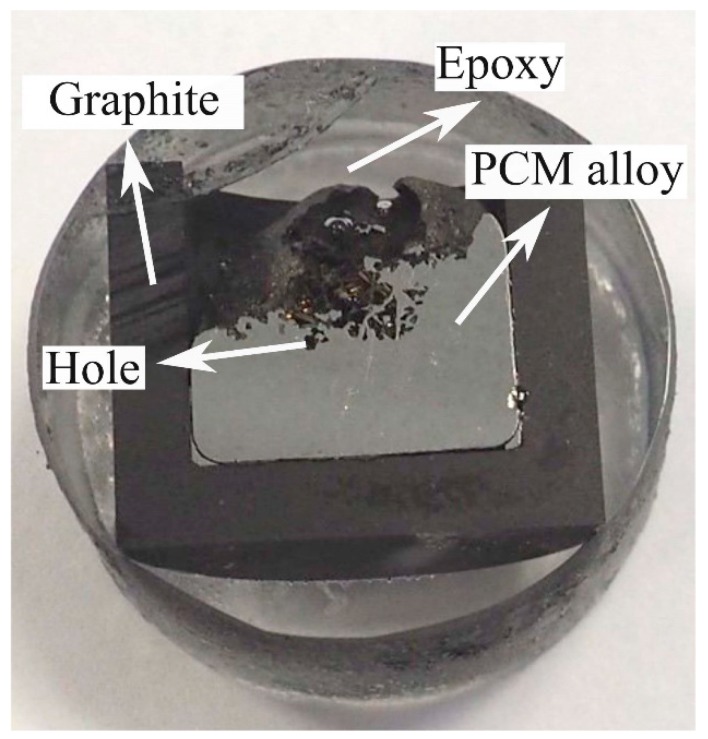
Photo of cross-sectioned graphite crucible with solidified Fe-26.38Si-9.35B alloy.

There are plenty of pores at the top and center positions of the Fe-26.38Si-9.35B ingot after melting/solidification process. The Fe-26.38Si-9.35B PCM alloy after four times thermal cycles at 1157 ± 100 °C in graphite crucible was used to perform a non-destructive analysis by computed tomography (CT) at the scanning rotation range of 0–360°. Four different angle positions of the sample are presented in Figure 17. Compared with the cross-section of the PCM alloy after the CT scan in Figure 18, it is seen that the solidified sample is not dense at the top and center positions of the sample as the small pores exist inside the sample. The PCM alloy close to the wall and bottom of the graphite crucible is however dense. Meanwhile, a crack line was observed at the bottom between the PCM alloy and graphite crucible, and this attests that the PCM alloy had a volume shrinkage during solidification process. 

**Figure 19:**
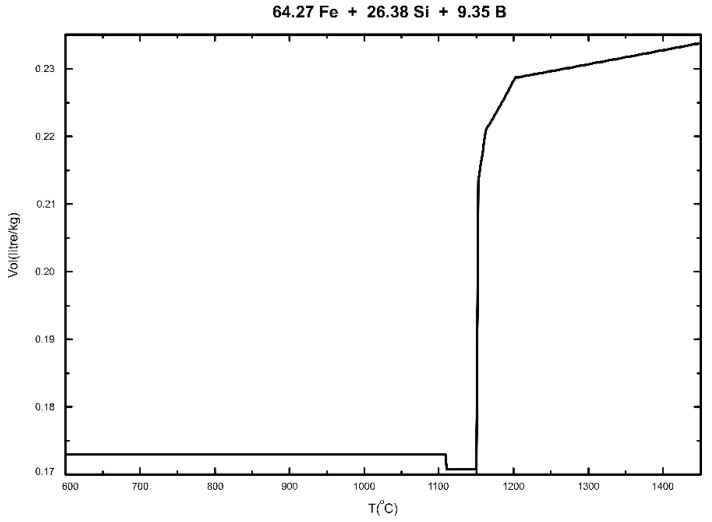
Calculated volume evolution of Fe-26.38Si-9.35B alloy with the decrease of temperature from 1450 °C to 600 °C.

The pores inside the Fe-26.38Si-9.35B alloy might arise from two causes [43]: (a) Shrinkage during solidification, and (b) exsolution of dissolved gases. The volume change of the Fe-26.38Si-9.35B alloy is calculated by FactSage software package and its commercial databases, as shown in Figure 19. It is obvious that the alloy volume decreases with the decrease of temperature. Notably, there is an evident volume shrinkage in the freezing point.

In the solidification process, the molten alloy close to the wall and bottom of the graphite is first solidified. The center part is finally solidified, which could not obtain enough molten alloy to supplement because of shrinkage. Hence, the pores are concentrated in the final solidification position. At the same time, the solubility of gas in the liquid state is normally higher than in the solid state [44]. Therefore, the gas precipitation may occur during solidification process due to the segregation of the gas elements. Examples of this is dissolved carbon and dissolved oxygen producing CO gas.

The pores in the Fe-26.38Si-9.35B alloy will destroy the metal continuity and decrease its corrosion resistance [45]. However, it can be controlled by reducing the dissolved gases content and decreasing the freezing rate [43]. In the TES, the optimal heat extraction would be from the bottom of the crucible. Thus, the pores will not affect the use of Fe-26.38Si-9.35B alloy as a PCM. 

## 5. Conclusions 

This work was conducted to investigate the application of Fe-26.38Si-9.35B eutectic alloy at 1157 ± 20 and 1157 ± 100 °C for 1–4 thermal cycles in the graphite crucible for a PCM material and the corresponding container development. The stability of the alloy and its interaction with the graphite container were theoretically and experimentally studied. The samples were investigated by EPMA-WDS, SEM-EDS, CT scan, XRD, and OM. The main conclusions are summarized as follow:

- The fusion enthalpy of the eutectic Fe-26.38Si-9.35B alloy is calculated to be 1250 kWh/m^3^. This value is higher than other possible PCMs candidates in the literature.
- Six different phases are found in the PCM alloy after solidification. FeSi, FeB, SiB_6_, and FeSiB_3_ are the main phases appeared in the sample. SiC and B_4_C particles are located at the edge of the treated alloy in graphite crucible. The new ternary phase, FeSiB_3_, is identified by wavelength dispersive X-ray spectroscopy analysis.
- Under conditions of this study, the penetration of molten PCM alloy into the graphite crucible is negligible and the alloy has no degradation effect on graphite container.
- Wetting experiment shows that the molten PCM alloy wets well with graphite substrate, for the low contact angle of 31°.The shrinkage of the Fe-26.38Si-9.35B alloy creates small amounts of pores at the top and center position of the solidified sample. No expansion upon solidification is promising for further use of this PCM alloy.
- Further study will focus on investigating the stability of Fe-26.38Si-9.35B alloy in other refractory materials (e.g. Si_3_N_4_, BN, and Al_2_O_3_) in the melting/solidification process. Other investigations, such as the fusion enthalpy of the Fe-26.38Si-9.35B alloy and the confirmation of the new FeSiB_3_ phase will be considered in subsequent works.

## Figures and Tables

**Table 1:** The thermal conductivity of the pure metal and its relative eutectic alloys.

Metal	Thermal Conductivity, λ (W/(m∙K))						
	30 °C	100 °C	300 °C	500 °C	700 °C	900 °C	1100 °C
Si[24]	142.2	97.4	57.7	40	29.8	28.9	28.7
Fe[25]	71.7	65.4	52.8	41.9	35.5	34.7	35.6
B[26]	26	–	–	–	–	–	–
Fe-26Si-9B	81	64.9	47.3	37.1	31.3	30.3	30.6

**Table 2:** The certified impurities in boron powder (ppm mass).

Sample	Boron
Fe	211
Cr	11
Si	16
Al	51
Zr	29
Mn	1

**Table 3:** The physical properties of the graphite and alumina crucibles.

Crucible	Property	Value	Unit
Graphite	Bulk	1.9	mg/m^3^
	Cumulative pore volume	0.052	m^3^/g
	Open porosity	10	vol %
	Radius of average open pores	1.4	µm
	Thermal conductivity	140	W/(m∙K)
Al_2_O_3_	Purity	99.8	%
	Operating temperature range	<1750	°C
	Permeability	Gas tight	-
	Water absorption	None	-

**Table 4:** Chemical composition of the master alloy analyzed by ICP-MS (mass %).

Sample		Si	Fe	B	Al	Mn
Fe-Si-B-1	Detected	26.68	56.47	8.11	0.067	0.21
	Normalized	29.15	61.69	8.86	0.073	0.23
Fe-Si-B-2	Detected	27.87	66.45	9.54	0.18	0.23
	Normalized	26.73	63.72	9.15	0.17	0.22
	Nominal composition	26.38	64.27	9.35		

**Table 5:** Fe, Si, and B contents in FeSi, FeSiB_3_, FeB, and SiB_6_ phases measured with WDS (at %).

Phase	Fe	Std. Dev.	Si	Std. Dev.	B	Std. Dev.
FeSi	45.5	±1.2	43.2	±1.1	11.3	±1.7
FeSiB_3_	22.0	±1.0	20.6	±1.4	57.3	±0.4
FeB	47.7	±0.9	0.23	±0.9	52.0	±0.1
SiB_6_	0.31	± 0.1	10.6	± 0.2	89.3	± 0.2
